# Promoting Self-Regulation in Health Among Vulnerable Brazilian Children: Protocol Study

**DOI:** 10.3389/fpsyg.2018.00651

**Published:** 2018-05-07

**Authors:** Luciana B. Mattos, Marina B. Mattos, Ana P. O. Barbosa, Mariana da Silva Bauer, Maina H. Strack, Pedro Rosário, Caroline T. Reppold, Cleidilene R. Magalhães

**Affiliations:** ^1^Education and Humanities Department, Federal University Health Sciences of Porto Alegre, Porto Alegre, Brazil; ^2^Department of Applied Psychology, School of Psychology, University of Minho, Braga, Portugal; ^3^Department of Psychology, Federal University Health Sciences of Porto Alegre, Porto Alegre, Brazil

**Keywords:** self-regulation, promotion health, school health program, healthy eating, oral health

## Abstract

The Health and Education Ministries of Brazil launched the Health in School Program (Programa Saúde na Escola - PSE) in 2007. The purpose of the PSE is two-fold: articulate the actions of the education and health systems to identify risk factors and prevent them; and promote health education in the public elementary school system. In the health field, the self-regulation (SR) construct can contribute to the understanding of life habits which can affect the improvement of individuals' health. This research aims to present a program that promotes SR in health (SRH). This program (PSRH) includes topics on healthy eating and oral health from the PSE; it is grounded on the social cognitive framework and uses story tools to train 5th grade Brazilian students in SRH. The study consists of two phases. In Phase 1, teachers and health professionals participated in a training program on SRH, and in Phase 2, they will be expected to conduct an intervention in class to promote SRH. The participants were randomly assigned into three groups: the Condition I group followed the PSE program, the Condition II group followed the PSRH (i.e., PSE plus the SRH program), and the control group (CG) did not enroll in either of the health promotion programs. For the baseline of the study, the following measures and instruments were applied: Body Mass Index (BMI), Simplified Oral Hygiene Index (OHI-S), Previous Day Food Questionnaire (PFDQ), and Declarative Knowledge for Health Instrument. Data indicated that the majority are eutrophic children, but preliminary outcomes showed high percentages of children that are overweight, obese and severely obese. Moreover, participants in all groups reported high consumption of ultraprocessed foods (e.g., soft drinks, artificial juices, and candies). Oral health data from the CI and CII groups showed a prevalence of regular oral hygiene, while the CG presented good oral hygiene. The implementation of both PSE and PSRH are expected to help reduce health problems in school, as well as the public expenditures with children's health (e.g., Obesity and oral diseases).

## Introduction

Health promotion for children has been receiving the attention of educators and researchers, and there has been a particular focus on oral health and eating habits (Yekaninejad et al., 2012; World Health Organization, 2016). According to the WHO report, the prevalence of obesity among children under the age of five has increased from 4.8 to 6.1% between 1990 and 2014; this entails that the number of children affected by this phenomenon has grown from 31 million to 41 million (World Health Organization, 2016).

Oral health involves health and well-being in an integral way, and despite being a preventable situation, oral diseases are considered endemic (Yekaninejad et al., 2012). Notwithstanding some improvements in oral health in developed countries, oral diseases such as dental plaque, gingival bleeding and dental caries are prevalent among schools worldwide, and are still considered public health problems (Yekaninejad et al., 2012).

In 2007, the Ministries of Health and Education from Brazil created the Health in School Program (Programa Saúde na Escola—PSE) with the aim of improving the school health system in Brazil (Brasil, 2007). The PSE is a school-based program built on the articulation of the educational and health systems to promote the education of health for the public schools students (Brasil, 2007). The main objective of the PSE is to detect risk factors and identify acts of preventive care while promoting the health of public elementary school students (e.g., assessing nutritional status, early incidence of hypertension and diabetes, caries control, visual and auditory acuity) (Brasil, 2015).

The social cognitive framework provides a relevant theoretical framework to the present study (Bandura, 1986). Social cognitive researchers have been specifically stressing the importance of people's agency as a construct of the assumption of one's personal responsibility in one's own behaviors (Bandura, 1986).

This has been the major focus of the research in the field of Self-Regulation in Health—SRH—(Bandura, 1986). Extant research has been focused on mapping the intervening variables in the process of building autonomy and responsibility (Zimmerman, 1986; Rosário et al., 2012a, 2015). Moreover, the design of intervention projects to promote self-regulatory processes as well as individuals' implications on their own health issues and the health outcomes have been receiving researchers' attention (Bandura, 2005; Silva and Pereira, 2012).

Self-regulation (SR) models have three customary sub-functions: (i) self-control of health-related behaviors and the cognitive and social conditions attached, (ii) adoption of objectives and strategies to achieve this self-control and (iii) self-reactivity, which involves self-motivating stimuli and social support networks that sustain healthy practices (Bandura, 2005). When focused on health, the SR construct can help build understanding of the processes involved in promoting lifelong habits. Thus, the promotion of SR is likely to improve individuals' health and personal well-being (Bandura, 2005). Extant literature has shown the efficacy of using the SR framework in health programs (e.g., the use of self-management strategies during the treatment of chronic diseases) (West et al., 1997; Fu et al., 2003; Clark et al., 2005) designed for improving health, decreasing the need for hospitalizations, and increasing the adherence to treatment (Haskell et al., 1994; West et al., 1997; Fu et al., 2003; Clark et al., 2005). However, research on the efficacy of school-based programs focused on promoting SR competencies in the health domain is still lacking (e.g., interventions targeting health eating and oral health) (Bandura, 2005).

### School health program—PSE programa saúde na Escola

The PSE is offered to Brazilian cities by the central government, and it involves the combined efforts of primary health care units and public schools (Brasil, 2007). The program has three components: (a) evaluation of the health conditions of the children and adolescents enrolled in public schools, (b) training on a set of activities of health promotion and risk prevention, and (c) the professional development and ongoing training of professionals from the educational and health systems (Brasil, 2015). To develop these actions, health professionals [nurses, community health workers (CHWs), dentists] conduct anthropometric evaluations (weight and height) and health assessments (healthy eating habits, oral health, and visual acuity) to students from all school grades (Brasil, 2015).

### Program to promote self-regulation in health (PSRH)

The PSRH is a program designed to promote the SR of health. The health contents of the PSRH are the same as those of the PSE (i.e. healthy eating and oral health habits). Moreover, the program is rooted on the social cognitive framework and the construct of SR (Rosário et al., 2012b). Both components are the theoretical ground for the story-tool, *Yellow's Trials and Tribulations*, which will be used to deliver the health contents and SR strategies (Rosário et al., 2017).

This story-tool aims to promote SR skills in children aged up to 10 years by teaching them learning strategies designed to accompany activities proposed by the PSRH. The book tells the story of the disappearance of the Yellow color from the Rainbow and the adventures of the other rainbow colors as they search for their missing friend (Rosário et al., 2012c). This story-tool addresses many practical examples of how children can use SR strategies to resolve their daily difficulties by increasing their autonomy in a responsible manner (Núñez et al., 2014; Rosário et al., 2017).

The present study should be interpreted as a response to three current issues: the health of Brazilian children, which in general is showing a negative trajectory despite the efforts of the PSE; the difficulties of children in developing systematic actions involving routine follow-up activities in PSE; and the lack of actions to promoting healthcare (Machado et al., 2015). To address the latter, this research aims to present a program that promotes SR in health. This program includes topics on healthy eating and oral health from the PSE; it is based on the social cognitive framework and uses story tools (Cabanach et al., 2009; Rosário et al., 2017) to train SRH in 5th grade students from the South of Brazil. The current program aims to promote the development of self-regulatory skills, which are considered essential for changing health-related behaviors (Bandura, 2005).

## Methods

**Figure 1:**
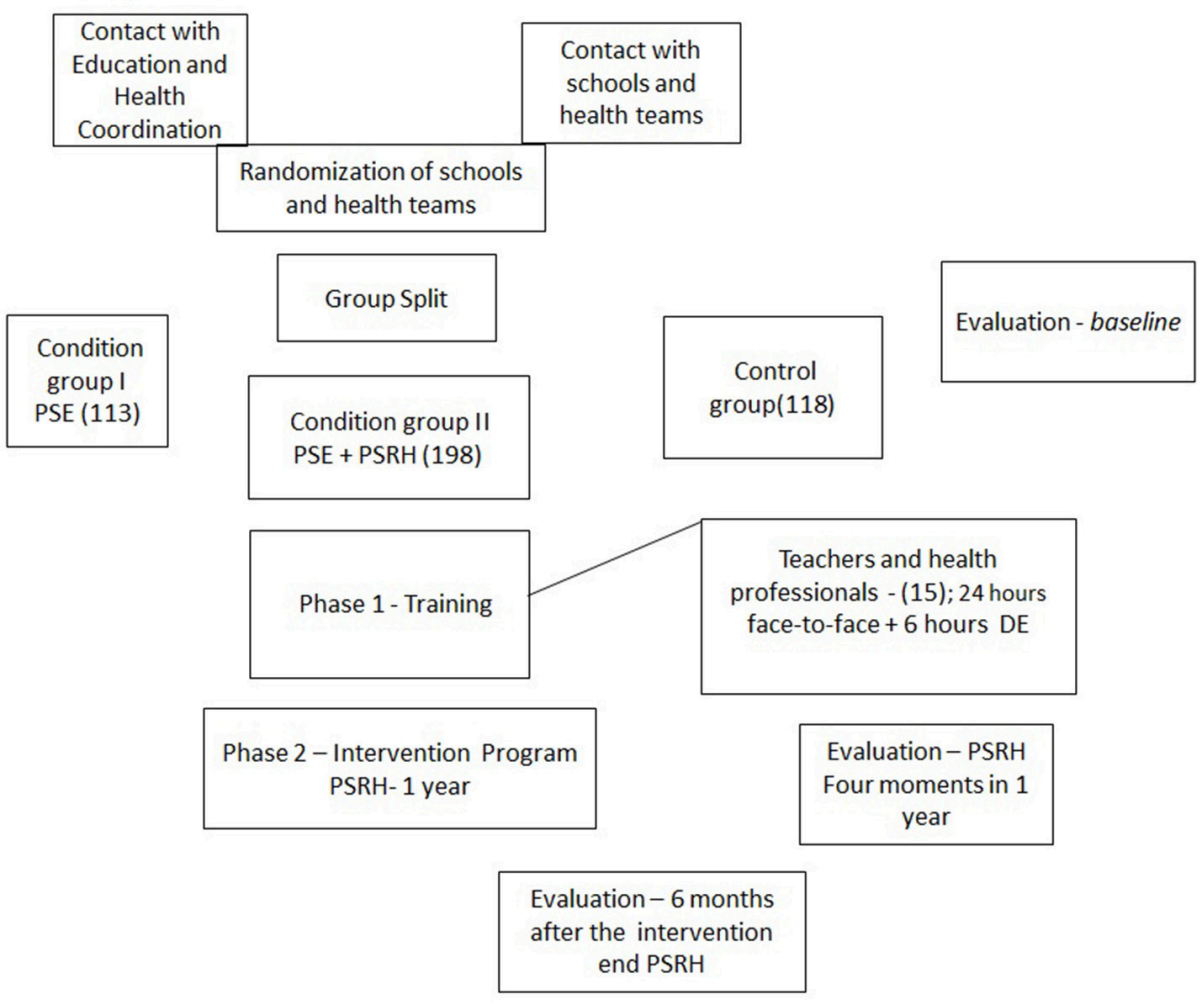
Promoting self-regulation in health among vulnerable Brazilian children: protocol study. Flow diagram for study procedures. PSE, Programa Saude na Escola; PSRH, Program to Promote Self-regulation in Health.

The current paper is a protocol study that describes a quasi-experimental study (Bedard et al., 2017). The development of the project will have two phases: Phase I: Deliver Training in Health Self-Regulation; and Phase II: Set an Intervention Program to Promote Self-regulation in Health (Figure 1).

### Contextualization of the study site

**Figure 2:**
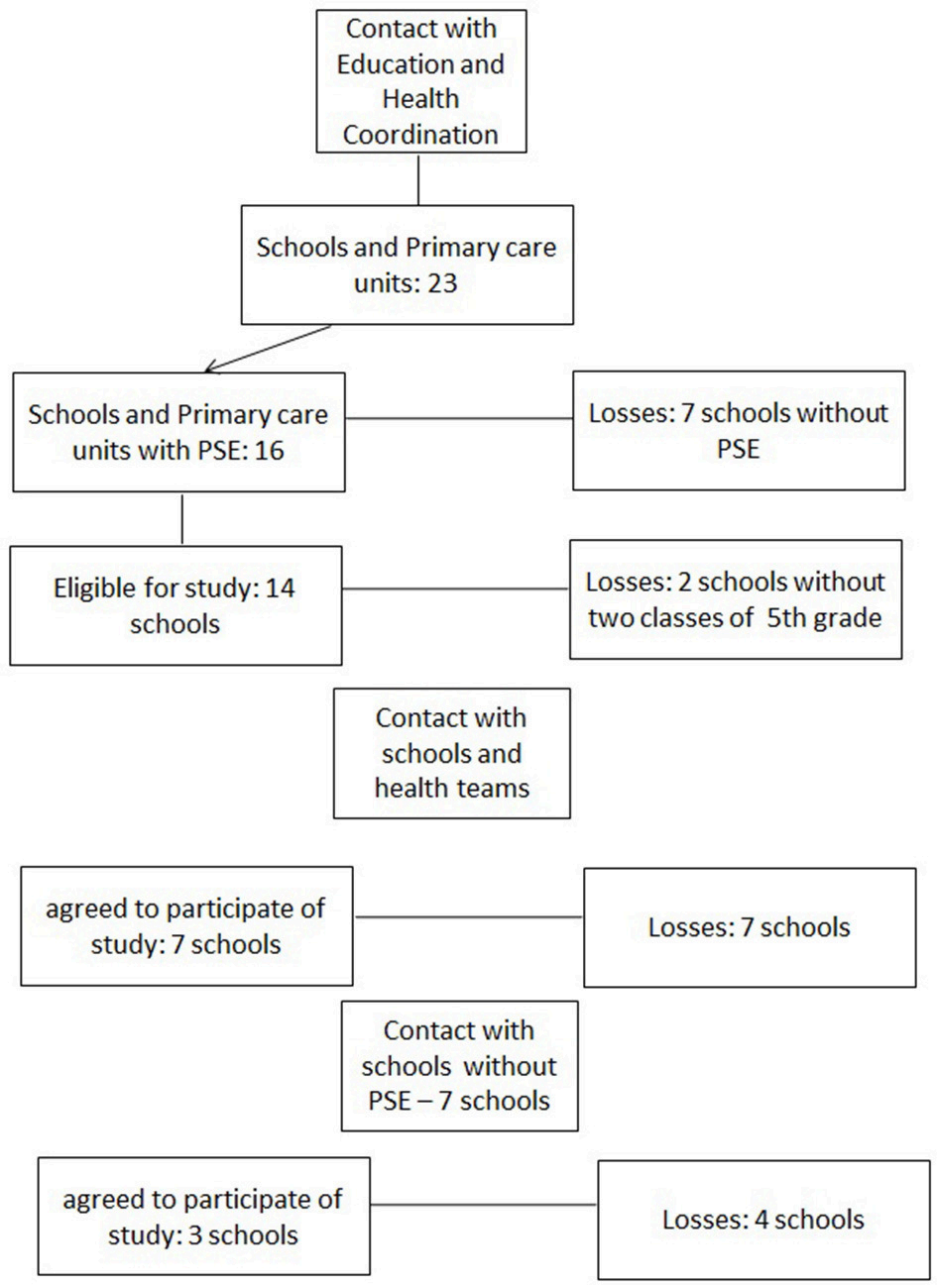
Promoting self-regulation in health among vulnerable Brazilian children: protocol study. Flow diagram to recruitment. PSE, Programa Saude na Escola.

The study will be conducted in a city in the south of Brazil (i.e., Sapucaia do Sul). This city has ~138,357 inhabitants and a lower average monthly income compared to neighboring cities in the region (IBGE, 2016). The high social vulnerability of the inhabitants of the city was the reason this town was chosen for the investigation. Sapucaia has 23 elementary schools working with Primary Health Care Units that employ doctors, nurses, nursing technicians, CHWs, dentists and oral health technicians (Sapucaia do Sul, 2016). Of these, 16 elementary schools are currently engaged with the PSE program. To engage in PSE, elementary schools and the primary health care units should form a dyad: each school has a health care unit partner to work with regarding health issues (Sapucaia do Sul, 2016). For the current study, the schools enrolled should have two classes in 5th grade. Only 14 out of 16 elementary schools in Sapucaia engaged in PSE met this criterion. All were invited to participate. Finally, seven dyads (school-health care unit) agreed to participate in the current investigation (response rate of 50%). Seven elementary schools which were not enrolled in PSE were contacted to participate as CG, but only three agreed (Figure 2). The reasons given by the schools for not enrolling in the research were not related with the nature or goals of the intervention, but with social and administrative limitations (e.g., general strikes that paralyzed public schools for several months, high workload and low salaries). The latter are examples that reflect the actual educational political environment in Brazil and stresses the relevance of developing research projects with vulnerable children, to help them with learning and health issues (Ribeiro, 2013; Casemiro et al., 2014).

### Recruitment and randomization

The school boards of 10 elementary schools agreed to participate. Participants were students and teachers from seven PSE elementary schools and health units, and three non-PSE schools. Finally, these schools were randomized into three groups: Control Group—CG—(eight classes)—schools not participating in PSE, Condition I—CI—(eight classes)—schools participating PSE; and Condition II—CII—(nine classes)—schools participating in the Phases I and II of the project.

### Study participants

Six hundred and twenty-five fifth grade students and their parents were contacted through face-to-face contacts (parent meetings and meetings with the teachers). Finally, 429 students [215 girls] were enrolled. These students are nested in 24 classes and their allocation to the three conditions was as follows: 8 classes with 118 students [62 girls] not enrolled in the PSE participated as CG; the remaining 17 classes were randomly split into two groups, 9 classes with 198 students [92 girls] in the CII, and 8 classes with 113 students [61 girls] in the CI.

#### Inclusion criteria

To be enrolled in this study, participants must meet the following criteria:

Teachers must teach a 5th grade class in a public elementary school;

Health professionals must be working in a primary care unit;

Students must be enrolled in the 5th grade in a public elementary school;

Parents/guardians: must be responsible for a child enrolled in a 5th grade class in a public elementary school;

All participants (parents and children) must be volunteers and must sign the Free and Informed Consent Term and Free and Informed Consent Term for parents/guardians authorizing their children to participate in the study. All subjects gave written informed consent in accordance with the Declaration of Helsinki.

#### Exclusion criteria

Potential participants who do not meet all the inclusion criteria, including 5th grade students with special educational needs that limit their cognitive autonomy, will be excluded from the study.

## Stepwise procedure

### Program rationale

The PSRH is grounded in the SR framework which describes the degree in which students are metacognitively, motivationally, and behaviorally engaged in their own learning processes (Zimmerman, 1989). SR processes may be described as open and dynamic processes proceeding through three main phases (i.e., forethought phase, the performance phase, and the self-reflection phase) (Zimmerman, 2002). The cyclical nature of this model aims to explain how students initiate, keep and control their behaviors, thoughts, and emotions toward specific goals. Motivational beliefs and task analysis are the two areas of the forethought phase, and they describe processes prior to learning efforts (e.g., goal setting, self-efficacy beliefs) (Rosário et al., 2013). The performance phase, describes the processes used by students' during learning. For example, self-instruction is a strategy that may help students focus their attention on homework assignments and eliminate distractors; and self-recording notes is a strategy that may help students self-monitor their performance (Zimmerman, 1989). Both strategies may facilitate self-control and self-observation, which are key components of the performance phase (Zimmerman, 2002). Lastly, the self-reflection phase describes methods intended to help students understand the processes that may have led to the outcomes and the reactions to these outcomes (Zimmerman, 1989). Self-judgments and self-reactions are the two areas of this last phase of the SR cycle (Zimmerman, 2002). For the purposes of the current work, the PLEE model, which is a SR model grounded on the model by Zimmerman (2002), will be used (Rosário et al., 2012a; Núñez et al., 2013). The abbreviation PLEE stands for the three phases that comprise the structure of the model: planning, task execution and evaluation (Pina et al., 2010). In this model, the logic and the cyclic movement is present at all times; during the planning phase, the execution and evaluation phases are still carried out (Rosário et al., 2017). For example, when children plan what they want to eat for lunch, they fulfill the execution phase by placing healthier foods in their lunch pack and they complete the self-reflection phase by evaluating their choices based on their learned experiences regarding nutrition.

### Phase 1—training in self-regulation in health

**Figure 3:**
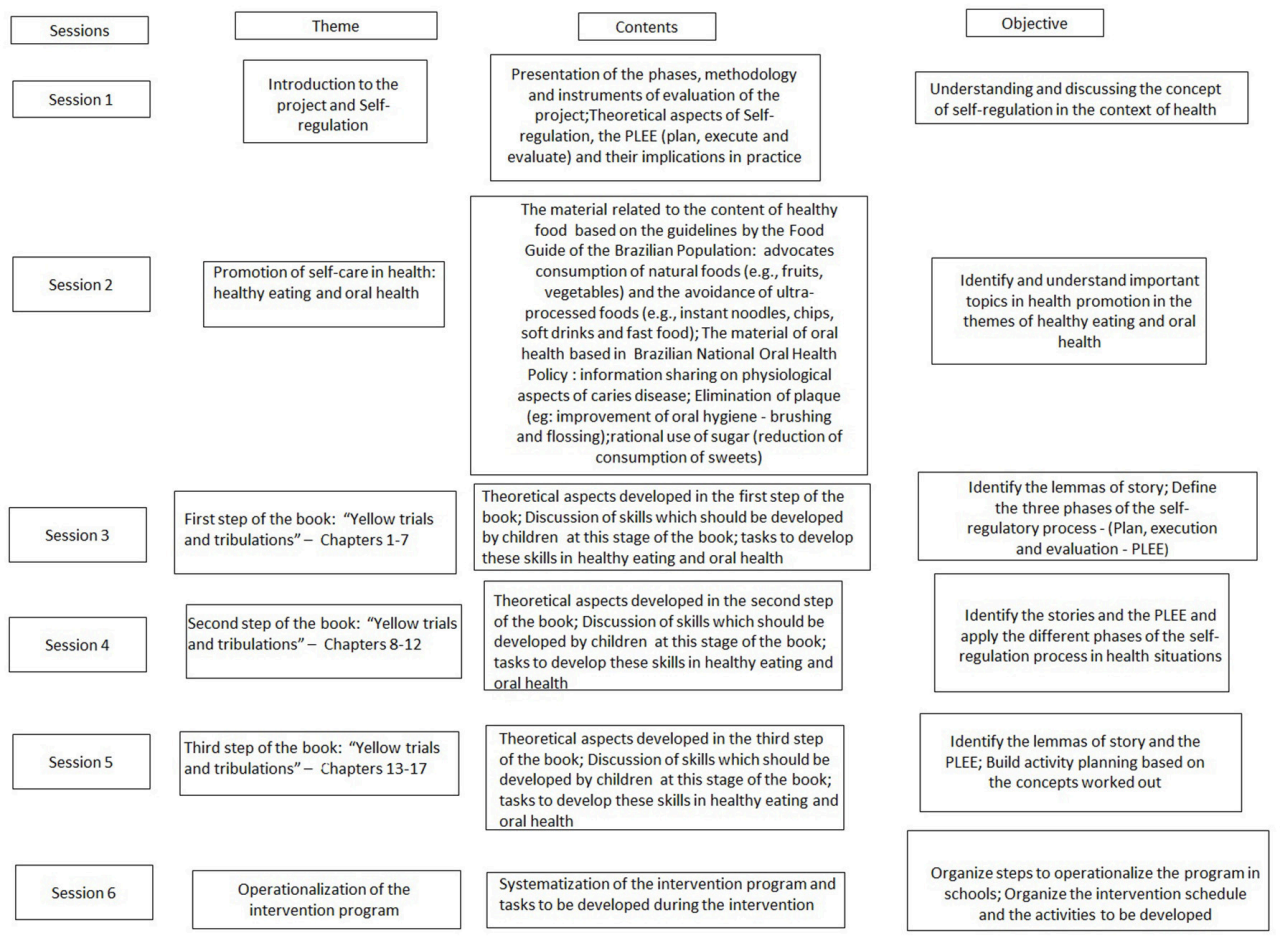
Promoting self-regulation in health among vulnerable Brazilian children: protocol study. Training Actives. PLEE, Plan, execution and evaluation.

During this stage, the training aimed to equip the participating professionals with the skills needed to conduct a program in SR focused on healthy eating and oral health habits. This training occurred in 2017 and was delivered by the authors and research assistants who have knowledge and skills in SR of health. The training duration was a total of 24 h, divided into 3 months (4 h sessions every 2 weeks of each month). The participants were health professionals (dentists, nurses, nursing technicians, CHWs) and 5th grade teachers of the CII schools. These sessions addressed the theoretical content related to SR, healthy eating, oral health and the chapters of the story-tool, *Yellow's Trials and Tribulations*, which was to be read and discussed (Rosário et al., 2012c). The sessions also included hands-on activities to build the support materials needed to work with children (e.g., drawings, worksheets, food maps;) (see Figure 3).

### Phase 2—intervention: program to promote self-regulation in health

The intervention program with children will be run by teachers and health professionals (CII) in 50-min biweekly sessions that will take place in class throughout the 2018 school year. During these sessions, the children will discuss the chapters of *Yellow's Trials and Tribulations* (Rosário et al., 2012b), one chapter per week, as well as the discussions and activities related to the topics of healthy eating and oral health (Table A1 in Appendix section).

The practice of storytelling has become an educational tradition that occurs in a variety of cultures. One of the reasons for using this technique may be related to the fact that stories are efficient ways of organizing knowledge (Rosário et al., 2017). When children become involved in a narrative, through reading or listening, they are likely to learn how to organize the information in a logical sequence (Alna, 1999).

Extant research indicates that discussion and interpretation of narratives may contribute to children's awareness of SR behaviors, which may be translated into their learning processes (Núñez et al., 2013). This process takes place through the development of vicarious learning by observing and expanding upon behaviors and expressions that help structure future modulations (Bandura, 1986; Schunk, 2000).

The *Yellow's Trials and Tribulations* story-tool is divided into three steps, each of them with specific goals and contents to be learned by children. By the end of the first step of the book (i.e., Chapters 1–7), the children are expected to be able to define the three phases of the SR process (PLEE) (Rosário et al., 2012a). After completing the second step (i.e., Chapters 8–12), the children are expected to be able to apply the PLEE model to situations of their everyday lives (Rosário et al., 2017). After completing the entire assigned reading, children are expected to be able to reflect on the importance of the SR strategies learned and transfer this knowledge to distinct domains of their lives (e.g., behavior in class, healthy food habits, time management; oral hygiene).

### Monitoring

The program will be monitored by researchers through case discussions and theoretical group meetings with teachers and health professionals which will occur during the biweekly visits to classes. Students in the three groups will be assessed five times throughout the year: before the initiation of the intervention program, 3 and 6 months later, at the end of the intervention, and 6 months post-intervention to check for the impact of the program on children's health.

### Ethics statement

This project was approved in the Ethics Committee of the Federal University of Health Sciences of Porto Alegre/Brazil—UFCSPA, n° 1.151.220 and through the Coordination for the Improvement of Higher Education Personnel (CAPES), Brazilian Federal agency for the Support and Evaluation of Graduate Education. The participation of children and parents, as well as their parents' consent was voluntary and unrewarded. Finally, informed consent was obtained from all parents/guardians regarding authorization of their children to participate in this study. All subjects (children, parents and consent of parents/guardians) gave written informed consent in accordance with the Declaration of Helsinki.

## Materials and equipment

### Instruments and measures

The effectiveness of the intervention will be assessed five times throughout the program. Ten self-reports (e.g., Food Preference Instrument, Students' Attitudes and Perceptions and Parents' Perceptions and Influences on the Health Instrument, Food Availability and Oral Health Instrument, Self-Regulation for Health Scale, Self-Efficacy for Health Scale) and two physical measures (e.g., BMI and OHI-S) will be used.

To characterize the baseline of this study, two questionnaires were used in 2016, before the start the program: Previous Day Food Questionnaire—*PDFQ;* and *Declarative Knowledge* and physical measures assessments (BMI e OHI-S) (Greene and Vermillion, 1964; World Health Organization and Multicentre Growth Reference Study Group, 2006; Penkilo et al., 2008; Assis et al., 2009; Wall et al., 2012).

#### Body mass index (BMI)

This anthropometric evaluation is one of the less invasive methods and has well established cutting techniques and points (Greene and Vermillion, 1964; World Health Organization and Multicentre Growth Reference Study Group, 2006; Brasil, 2008). It is the most commonly used method in interventions that focus on obesity prevention (Kamath et al., 2008; Friedrich et al., 2012; Bogart et al., 2014). Validation studies of this instrument are limited in quantity. An internal and external validation study showed valid estimates regarding the weight of the subjects evaluated by this scale (Deurenberg et al., 1991). The data were obtained by determining the weight and height of the students by using an electronic scale and stadiometer for each measurement respectively. The devices were calibrated in the Nutrition Laboratory of the Federal University of Health Sciences of Porto Alegre and operated by nutrition researchers from these labs. To guarantee the reliability of the measures, all researchers followed the same protocol throughout evaluations. To classify the nutritional status of the schoolchildren, the data on height/age z scores (E/I) and body mass/age index (BMI/I) were used, following the standards of the World Health Organization and Multicentre Growth Reference Study Group (2006). We used the following cut-off points for E/I: z <-3 (low height), −3 ≤ z <−2 (low height), z ≥ −2 (height suitable); the following cut-off points were used for BMI/3 (thinness): −3 ≤ z <−2 (severe thinness), −2 ≤ z < +1 (eutrophic/normal), +1 ≤ z < +2 (overweight), +2 ≤ z < +3 (obesity), Z ≥ + 3 (severe obesity) (World Health Organization and Multicentre Growth Reference Study Group, 2006).

#### Simplified oral hygiene index

OHI-S is a classic measure used to determine the impact of health education on oral hygiene (Greene and Vermillion, 1964; Silveira et al., 2002; Cardoso et al., 2011; Scopel et al., 2011). To assess the oral health condition the index OHI-S was used. This index measures plaque accumulation on six dental surfaces (16, 11, 26 and lingual vestibular of 31, 36, 46) (Greene and Vermillion, 1964). Each surface is evaluated according to the scores on a scale from 0 to 3: 0—The surface is free of plaque; 1—Less than 1/3 of the tooth covered per plate; 2—Between 1/3 and 2/3 of the tooth is covered per plate; 3—More than 2/3 of the tooth is covered per plate. The final result of this evaluation is obtained by dividing the sum of the values by the number of surfaces evaluated (Greene and Vermillion, 1964). The values obtained indicate the oral health on a range between good and poor hygiene: values from 0.0 to 0.6 indicate good hygiene, values from 0.7 to 1.8 indicate regular hygiene, and values from 1.9 to 3.0 indicate poor hygiene (Greene and Vermillion, 1964). Oral Hygiene Index (OHIS) is recognized to be a useful index for evaluation of dental health education in public school systems. Literature has been stating that OHIS is a sensitive method that can be used to evaluate oral hygiene of population groups with confidence (Greene and Vermillion, 1964; Mbawalla et al., 2010).

#### Previous day food questionnaire (PFDQ)

The PFDQ is an illustrated instrument that seeks information from schoolchildren about the food they consumed on the day prior (Assis et al., 2009). The meals were arranged in chronological order: breakfast, mid-morning snack, lunch, afternoon snack, dinner, and evening snack (Assis et al., 2009). Each meal was illustrated by 21 individual foods and some food groups: dry beans, rice, milk, coffee with milk, chocolate milk, cheese, yogurt, beef or poultry, pasta, bread or crackers, French fries, pizza or hamburger, leafy vegetables, starchy vegetables, vegetable soup, fruits, sweets, chips, fish/sea foods, soft drinks, and fruit juices (Assis et al., 2009). The reliability of this instrument to assess the foods consumed was 70.2% and the non-consumed food was 96.2%. In Brazil, studies were also conducted using multivariate logistic regression. Data showed that the frequency of discordance ranged from 3.7 to 39.6% (Assis et al., 2009). Children in 5th grade classes will complete this questionnaire three times a week in class and at home, the latter with the parents/guardians acting as responsible mediators.

#### Declarative knowledge for health instrument (DKH)

In this study, the Declarative Knowledge for Health (DKH) is an adaptation of the Nutritional Monitoring questionnaire (Penkilo et al., 2008; Assis et al., 2009). Questions aim to evaluate children's knowledge about healthy eating and oral health (Penkilo et al., 2008; Wall et al., 2012). This instrument consists of 20 questions (10 questions for each theme). In the current study, the coefficient of Alpha of Cronbach indicated an internal consistency of 0.71 for healthy eating and 0.76 for oral health.

## Proposed analysis

Data will be analyzed with linear mixed models using IBM SPSS Statistics version 22 with alpha levels set at *p* ≤ 0.05. It is expected that at the end of the intervention significant differences will occur with an increase in SRH, self-efficacy, and declarative knowledge in both domains (healthy eating and oral health) for CII in relation to the other two groups (CI and CG). Moreover, in relation to healthy eating, at the end of the program it is expected that the consumption of fruits and vegetables may increase, and the consumption of ultraprocessed foods may decrease; consequently a reduction in obesity and overweightness is expected. While focusing on oral health, at the end of the intervention it is expected for the CII group to show better brushing and care in relation to oral health and consequently an improvement in the health situation reflected in dental plaque reduction (and possible oral diseases prevention). These hypotheses will be studied through the intragroup analysis and intergroup with ANOVA of repeated measures, during the five moments of evaluation of the program. Differences between conditional and control groups at baseline were examined using Chi-square test (χ^2^) of heterogeneity comparing the proportions between the groups, significance levels were set at *p* < 0.05, and descriptive analysis of frequencies, mean, and standard deviation were done.

## Anticipated results

### Baseline

The preliminary outcomes show the first application of PFDQ, DKH, and physical measurement evaluation (BMI and OHI-S) (Greene and Vermillion, 1964; Penkilo et al., 2008; Assis et al., 2009; Wall et al., 2012; World Health Organization, 2016). These data were collected prior to the beginning of the project in order to characterize the baseline.

In terms of the anthropometric data, 429 students (198 from the CII, 113 from the CI and 118 from the CG) participated in data collection. The mean age of participants was 10.61 years (*SD*−1.06). When assessing the nutritional status of children, according to z-score of BMI/weight and height/ age, the researchers observed that there were no differences between groups (World Health Organization and Multicentre Growth Reference Study Group, 2006). The prevalence of eutrophic/normal children with adequate heights for their age was 96 (24.5%), 57 (14.5%), and 67 (17.1%), respectively. However, there were high percentages of overweight, obesity and severe obesity in all groups (Table 1).

**Table 1:** Data of BMI/weight and Height for age, Z-score (Z) (World Health Organization and Multicentre Growth Reference Study Group, 2006).

**BMI CLASSIFICATION DATA**	**CII (198)**	**CI (113)**	**CG (118)**	**Total (429)**
Eutrofhy/height suitable for age	94 (24.5%)	57 (14.5%)	67 (17.1%)	218 (50.8%)
Eutrofhy/low height for age	2 (1.07%)	–	–	2 (0.5%)
Overweight/height suitable for age	47 (12.0%)	30 (7.7%)	19 (4.8%)	96 (22.4%)
Obesity/ height suitable for age	25 (6.4%)	18 (4.6%)	24 (6.1%)	67 (15.7%)
Severe obesity/height suitable for age	10 (5.0%)	5 (4.4%)	4 (3.4%)	19 (4.4%)
Thinness/height suitable for age	4 (1.0%)	2 (0.5%)	2 (1.7%)	8 (1.9%)
Severe thinness/low height for age	–	–	2 (1.7%)	2 (0.4%)

*BMI, Body Mass Index; CII, condition II group; CI, condition I group; CG control group*.

Regarding the Declarative Knowledge in health, we did not observe statistically significant differences in the number of correct answers between healthy eating and oral health in the participating groups (the level of significance was 0.05). Focusing on the knowledge related to the theme of healthy eating, only two items (3 and 5) presented significant differences in the number of correct answers between the groups [item 3: χ^2^ = 7.20, *p* = 0.027 and Item 5: χ^2^ = 12.38, *p* = 0.002]. These questions relate to fruits and vegetables (e.g., “It is necessary to eat fruits and vegetables but not all days”) and biscuits with sugar (e.g., “Biscuits with sugar are industrial foods”). In the two items, the CI Group (schools enrolled in the PSE), had more correct answers than the other two groups. This may indicate that this content knowledge had already been addressed during the PSE sessions run by the primary care teams (Brasil, 2015) as well as by the teachers in science classes (Brasil, 2016).

Regarding the oral health data, a difference was found between the groups. Globally, the CII and CI groups showed a prevalence of regular oral hygiene, 103 (25.0%) and 58 (14.1%), respectively; while the CG presented a good oral hygiene, 67 students (16.3%), as shown in Table 2. This good oral hygiene status of the CG may be due to more frequent and adequate brushing techniques used. Regarding the oral health topic, three items presented differences between the groups in terms of the number of correct answers (item 1: χ^2^ = 9.15, *p* = 0.010; Item 8: χ^2^ = 8.50, *p* = 0.014 and item 9: χ^2^ = 7.35, *p* = 0.025). These items regard caries disease (e.g., “Carie is not caused by bacterias”), toothbrush cleanliness (e.g., “I need to change my toothbrush once per year”) and bacterial plaque (e.g., “Bacterial plaque can be removed by brushing teeth”). The highest number of correct answers was obtained by CI and CII groups.

**Table 2:** Data Simplified Oral Hygiene Index – OHI-S (Greene and Vermillion, 1964).

**OHI-S DATA**	**CII (198)**	**CI (113)**	**CG (118)**
Poor	8 (1.9%)	16 (3.9%)	1 (0.2%)
Regular	103 (25.0%)	58 (14.1%)	50 (12.1%)
Good	71 (17.2%)	38 (9.2%)	67 (16.3%)

*CII, condition II group; CI, condition I group; CG control group*.

The outcomes related to the previous day food questionnaire (PDFQ) describe the food that was consumed on 3 days of the week (1 weekend day and 2 weekdays), six meals per day (breakfast, mid-morning snack, lunch, afternoon snack, dinner, and evening snack) (Assis et al., 2009). Data were organized and analyzed as follows: the 21 foods depicted in the PDFQ were collected in ten large food groups in accordance to the Food Guide of the Brazilian Population, which describes the food groups that should have highest prevalence for each meal (Table 3) (Assis et al., 2009; Brasil, 2014). Moreover, the students in the three groups reported the food they had consumed within the previous 3 days prior to the questionnaire. The food that was most commonly consumed for breakfast and mid-morning snack meals included the following: dairy products (milk, yogurt, cheese, chocolate), cereals (bread, wafer, rice, pasta), and fruit (fruit and fruit juices) groups. Another observation worth noting is that for snacks, the children tended to consume candies (cakes and sweets in general), soft drinks (and artificial juices), and chips (Currie et al., 2012). The most frequent food groups reported in lunch and dinner were cereals (bread, crackers, rice, pasta, and potatoes), proteins (meats in general, eggs) and soft drinks (Table 4). It should be noted that the vegetable groups had a lower prevalence than that of soft drinks (and artificial juices). The afternoon and evening snacks included the following as main food groups: cereals, milk and dairy products, with an emphasis on fast food in the evening (e.g., chips, hamburgers, pizza, and ultra-processed snacks) (CGI−26%, CGII−18%, CG−27%, see Table 4) (Brasil, 2014).

**Table 3:** Food Consumption information– PFDQ (Assis et al., 2009).

**Meals**	**n° meals/week**					**Food groups consumed**			
	**CI**	**CII**	**CG**	**CI**		**CII**		**CG**	
Breakfast	296	422	295	Milk/derivatives	227 (76.68%)	Milk/derivatives	307 (72.74%)	Milk/derivatives	265 (89.83%)
				Cereals	218 (73.64%)	Cereals	267 (63.27%)	Cereals	226 (76.61%)
				Fruits	62 (20.94%)	Fruits	58 (13.74%)	Fruits	44 (14.91%)
				Candies	19 (6.41%)	Candies	48 (11.37%)	Candies	22 (7.45%)
				Soft drinks	19 (6.41%)	Soft drinks	36 (8.53%)	Soft drinks	17 (5.76%)
Mid-morning snack	239	318	220	Milk/derivatives	109 (45.60%)	Milk/derivatives	117 (36.79%)	Milk/derivatives	71 (32.27%)
				Cereals	91 (38.07%)	Cereals	87 (27.35%)	Cereals	70 (31.81%)
				Fruits	75 (31.38%)	Fruits	78 (24.52%)	Fruits	61 (27.72%)
				Candies	44 (18.41%)	Candies	53 (16.66%)	Candies	60 (27.27%)
				Soft drinks	42 (17.57%)	Soft drinks	50 (15.72%)	Soft drinks	43 (19.54%)
				Salty snacks (chips)	29 (12.13%)	Salty snacks (chips)	45 (14.15%)	Salty snacks (chips)	33 (15%)
Lunch	335	527	347	Cereals	295 (88.05%)	Cereals	437 (82.92%)	cereals	316 (91.06%)
				Proteins	233 (69.55%)	Proteins	355 (67.36%)	Proteins	267 (76.94%)
				Legume	188 (56.11%)	Legume	269 (51.04%)	Legume	213 (61.38%)
				Soft drink	114 (34.92%)	Soft drink	156 (29.60%)	Soft drink	97 (27.95%)
				Vegetables	88 (26.26%)	Vegetables	133 (25.23%)	Vegetables	88 (25.36%)
				Fruits	50 (14.92%)	Fruits	82 (15.55%)	Fruits	54 (15.56%)
				Fast food	32 (9.55%)	Fast food	35 (6.64%)	Fast food	29 (8.35%)

*PFDQ, Previous Day Food Questionnaire; CI, condition I group; CII, condition II group; CG control group*.

**Table 4:** Food Consumption information– PFDQ (Assis et al., 2009).

Afternoon snack	320	487	322	Cereals	176 (55%)	Cereals	244 (50.10%)	Cereals	155 (48.13%)
				Milk/derivates	150 (46.87%)	Milk/derivates	214 (43.94%)	Milk/derivates	137 (42.54%)
				Candies	81 (25.31%)	Candies	125 (25.66%)	Candies	98 m(30.43%)
				Fruits	77 (24.06%)	Fruits	96 (19.71%)	Fruits	92 (28.57%)
				Soft drinks	72 (22.5%)	Soft drinks	92 (18.89%)	Soft drinks	78 (24.22%)
				Salty snack (chips)	51 (15.93%)	Salty snack (chips)	56 (11.49%)	Salty snack (chips)	43 (13.35%)
				*Fast food*	28 (8.75%)	*Fast food*	24 (4.92%)	*Fast food*	29 (9%)
Dinner	321	488	328	Cereals	251 (78.19%)	Cereals	343 (70.28%)	Cereals	259 (78.96%)
				Proteins	189 (58.87%)	Proteins	240 (49.18%)	Proteins	187 (57.01%)
				Legume	161 (50.15%)	Legume	178 (36.47%)	Legume	150 (45.73%)
				Soft drinks	95 (29.59%)	Soft drinks	134 (27.45%)	Soft drinks	91 (27.74%)
				Vegetables	86 (26.79%)	Vegetables	112 (22.95%)	Vegetables	79 (24.08%)
				Fruits	41 (12.77%)	Fruits	77 (15.77%)	Fruits	46 (14.02%)
				*Fast food*	37 (11.52%)	*Fast food*	72 (14.75%)	*Fast food*	47 (14.32%)
				Milk/derivates	21 (6.54%)	Milk/derivates	31 (6.35%)	Milk/derivates	16 (4.87%)
Evening snack	246	313	230	Milk/derivates	87 (35.36%)	Milk/derivates	104 (33.22%)	Milk/derivates	77 (33.47%)
				Candies	74 (30.06%)	Candies	91 (29.07%)	Candies	72 (31.30%)
				Fruits	69 (28.04%)	Fruits	68 (21.72%)	Fruits	64 (27.82%)
				Cereals	61 (24.79%)	Cereals	68 (21.72%)	Cereals	58 (25.21%)
				Soft drinks	61 (24.79%)	Soft drinks	64(20.44%)	Soft drinks	43(18.69%)
				Salty snacks (chips)	24 (9.75%)	Salty snacks (chips)	24 (7.66%)	Salty snacks (chips)	27 (11.73%)
				*Fast food*	26 (10.56%)	*Fast food*	18 (5.75%)	*Fast food*	20 (8.69%)

*PFDQ, Previous Day Food Questionnaire*.

## Discussion

The data found are consistent with general data from the Brazilian population-based studies involving school children: findings show a low rate of nutritional deficits and an increase in overweightness and obesity (Ruiz et al., 2009). In a study involving 3,387 school children between the ages of seven and ten, in the public school system of Rio de Janeiro, data showed that the students had a prevalence of eutrophy/normal weight, followed by overweightness and obesity (Anjos et al., 2003). Percentages of overweightness and obesity were identified in all groups as well as unhealthy eating habits such as the following: the consumption of soft drinks and artificial juices for almost all meals; the consumption of snacks, candies and fast food for breakfast; and the higher consumption of soft drinks and candies over vegetables. These eating behaviors, particularly low consumption of fruits and vegetables and high consumption of sweets, candies, and beverages rich in sugar and fats, have been indicated by literature as significant risk factors for overweightness and obesity (Neutzling et al., 2007; Tarek et al., 2008; Bertin et al., 2010).

Declarative knowledge describes how people define their knowledge. It is comprised of the process of information and how people understand concepts (Rosário et al., 2017). Data on declarative knowledge about healthy eating have indicated that children maintain unhealthy eating habits even though they have knowledge about healthy eating (Gaspar et al., 2014). This may suggest that children have difficulties in regulating their eating behavior (Anderson et al., 2007). Therefore, school interventions in this topic may wish to develop a set of self-regulatory skills needed to develop healthy eating habits (Anderson et al., 2007).

Data for oral health were also gathered from all the groups enrolled. The students in the groups that participated previously in PSE showed lower oral hygiene than the students in the control group; the latter will not participate in school activities systematically implemented on this topic. This finding was unexpected because the students in Condition I and Condition II participated in the PSE activities and had several opportunities to practice oral care, while students in the Control group did not have this training.

This good practice prevents the generation of plaque on the surfaces of the teeth as well as, overall, oral disease. Prior research indicates that adequate oral hygiene is related to the absence of caries in school children (Anagnostopoulos et al., 2011). Another study pointed out that educational-preventive activities with school children and preschoolers, even for a short period, may be effective for reducing visible plaque and gingival bleeding (Barreto et al., 2013). However, the current study findings suggest that to maintain positive results, the intervention program must be long-term (Pauleto et al., 2004).

In recent years, the number of oral health programs offered to school children has increased. However, the programs still hold an approach more focused on medicalized treatments than on educational promotion, for example stressing students agent role in their health (Pauleto et al., 2004). Notwithstanding, even school-based programs with an educational approach lack opportunities to discuss and reflect on health behaviors and improve SR. The SR practices are important because they are likely to promote autonomy and instigate good oral health care (Pauleto et al., 2004). Initial data allows us to conclude that children's participation in the PSE intervention for 4 years (from 1st to 4th grade) and the acquisition of knowledge on healthy eating and oral health is not enough to promote and sustain good health habits. Data seem to be indicating that besides the health knowledge learnt with the PSE intervention, children may need intentional educational training on SR to help them change their health behavior. Present findings indicate the need to expand PSE interventions while emphasizing the development of SR competences for self-management and self-control of health-related behaviors. This training is expected to help children set goals and display strategies to achieve, and afterwards sustain, good health practices.

## Limits

The possible limitations of this study may stem from the restricted numbers of participants enrolled and the design, as follows: the study was restricted to only one city with their schools and health services; data collection may be impacted by the possible loss of participation, especially considering that this investigation will be run throughout a school-year and per losses of the schools who did not agree to participate in the study.

The biweekly monitoring of the Condition II, the training program and the guidance of materials are among the several strategies expected to help solve these possible external situations (e.g., stoppages due to teachers strikes, withdrawal of study participation, transfers of students and school teachers).

## Conclusions

The present study is expected to contribute to understand the impact of a health public policy implemented all over the country. The findings are expected to help reinforce the importance of the multidisciplinary action of health and education professionals, and this interdisciplinary articulation favors health promotion. The PSRH is designed to respond to this call. This program aims to equip the students with the skills and knowledge to improve their self-care habits, their organization in their daily life and their overall autonomy. Moreover, it can be further used as a tool to train teachers and health professionals so that they help students throughout the stages and processes of SR (e.g., PLEE) (Núñez et al., 2013). It is hoped that the training provided on SRH for health professionals and teachers, and the implementation of the PSRH in schools help children to become more autonomous and responsible regarding their self-care on healthy eating and oral health. In consequence, PSRH is expected to help reduce children's health problems, as well as public expenditures with children's health (e.g., obesity and oral diseases).

## Author contributions

LM, CM, PR, MB, and MS: contributed to the build design and conduct the training of the Program; MM and AB: contributed to the organization and analysis of data; CR, CM, and PR: contributed to the writing, discussion, and approval of the manuscript.

### Conflict of interest statement

The authors declare that the research was conducted in the absence of any commercial or financial relationships that could be construed as a potential conflict of interest.

## Appendix

**Table A1:** Examples of activities 1 month in three groups.

	**Activities**	**Periodicity**	**Methodology**	**Responsible**
CGI	Educational activity on healthy eating	Monthly	Lecture	Health care professional
	Educational activity on oral health	Monthly	Lecture	Dentist or oral health technician
CGII	Educational activity on healthy eating	Monthly	Lecture	Health care professional
	Educational activity on oral health	Monthly	Lecture	Dentist or oral health technician
	Reading chapter 1 and reflection;	Weekly	Collective reading and discussion in a large group	5th grade teacher—trained by PSR
	“Colors” activity; Identification with the colors of the students' history and characteristics	Weekly	Discussion in small and large groups	5th grade teacher and health professional—trained by PSR
	Reading chapter 2 and reflection;	Weekly	Collective reading and discussion in a large group	5th grade teacher—trained by PSR
	Activity: “Order of things”—reflect on order of things in life, health care (healthy eating and oral health)	Weekly	Discussion in a large group	5th grade teacher and health professional—trained by PSR
	- Reading chapter 3 and reflection	Weekly	Collective reading and discussion in a large group	5th grade teacher—trained by PSR
	- Activity: ”What would happen if …"—reflect on the role of all (healthy eating and oral health)	Weekly	Discussion in a large group	5th grade teacher and health professional—trained by PSR
	Reading chapter and reflection	Weekly	Collective reading and discussion in a large group	5th grade teacher—trained by PSR
	Activity: Planning—Reflect on the importance of planning actions (health care)	Weekly	Reading and working in small groups	5th grade teacher and health professional—trained by PSR
CG	Curricular school activities	weekly	Exhibition classes; Group discussions	5th grade teacher—trained by PSR
